# Indicative Marker Microbiome Structures Deduced from the Taxonomic Inventory of 67 Full-Scale Anaerobic Digesters of 49 Agricultural Biogas Plants

**DOI:** 10.3390/microorganisms9071457

**Published:** 2021-07-07

**Authors:** Julia Hassa, Johanna Klang, Dirk Benndorf, Marcel Pohl, Benedikt Hülsemann, Torsten Mächtig, Mathias Effenberger, Alfred Pühler, Andreas Schlüter, Susanne Theuerl

**Affiliations:** 1 Center for Biotechnology (CeBiTec), Bielefeld University, Universitätsstrasse 27, 33615 Bielefeld, Germany; jhassa@cebitec.uni-bielefeld.de (J.H.); puehler@cebitec.uni-bielefeld.de (A.P.); aschluet@cebitec.uni-bielefeld.de (A.S.); 2 Department Bioengineering, Leibniz Institute for Agricultural Engineering and Bioeconomy, Max-Eyth-Allee 100, 14469 Potsdam, Germany; jklang@atb-potsdam.de; 3 Bioprocess Engineering, Otto von Guericke University, Universitätsplatz 2, 39106 Magdeburg, Germany; benndorf@mpi-magdeburg.mpg.de; 4 Bioprocess Engineering, Max Planck Institute for Dynamics of Complex Technical Systems, Sandtorstraße 1, 39106 Magdeburg, Germany; 5 Microbiology, Anhalt University of Applied Sciences, Bernburger Straße 55, 06366 Köthen, Germany; 6 Biochemical Conversion Department, DBFZ Deutsches Biomasseforschungszentrum Gemeinnützige GmbH, Torgauer Straße 116, 04347 Leipzig, Germany; marcel.pohl@dbfz.de; 7 The State Institute of Agricultural Engineering and Bioenergy, University of Hohenheim, Garbenstraße 9, 70599 Stuttgart, Germany; benedikt.huelsemann@uni-hohenheim.de; 8 Institute of Agricultural Engineering, Kiel University, Max-Eyth-Str. 6, 24118 Kiel, Germany; tmaechtig@ilv.uni-kiel.de; 9 Institute for Agricultural Engineering and Animal Husbandry, Bavarian State Research Center for Agriculture, Vöttinger Str. 36, 85354 Freising, Germany; mathias.effenberger@LfL.bayern.de

**Keywords:** anaerobic digestion, biogas microbiome, taxonomic profiling, 16S rRNA gene amplicon sequencing, NMDS, indicative taxa, Pearson correlations

## Abstract

There are almost 9500 biogas plants in Germany, which are predominantly operated with energy crops and residues from livestock husbandry over the last two decades. In the future, biogas plants must be enabled to use a much broader range of input materials in a flexible and demand-oriented manner. Hence, the microbial communities will be exposed to frequently varying process conditions, while an overall stable process must be ensured. To accompany this transition, there is the need to better understand how biogas microbiomes respond to management measures and how these responses affect the process efficiency. Therefore, 67 microbiomes originating from 49 agricultural, full-scale biogas plants were taxonomically investigated by 16S rRNA gene amplicon sequencing. These microbiomes were separated into three distinct clusters and one group of outliers, which are characterized by a specific distribution of 253 indicative taxa and their relative abundances. These indicative taxa seem to be adapted to specific process conditions which result from a different biogas plant operation. Based on these results, it seems to be possible to deduce/assess the general process condition of a biogas digester based solely on the microbiome structure, in particular on the distribution of specific indicative taxa, and without knowing the corresponding operational and chemical process parameters. Perspectively, this could allow the development of detection systems and advanced process models considering the microbial diversity.

## 1. Introduction

The production of biogas by anaerobic conversion of organic matter from various residues is a worldwide applied technology that can be integrated into sustainable bioeconomic concepts [1,2,3]. The production of biogas offers a number of benefits such as residue treatment [3], energy generation (electricity, heat and fuels) [3,4,5], mitigation of greenhouse gas emissions (in particular methane and nitrous oxide) [6,7], production of high-value fertilizers [8,9,10] as well as closing nutrient cycles [11,12].

However, in the past two decades, more than 9500 biogas plants in Germany have mainly been operated with energy crops (48.9%) and livestock manure (44.5%) [13], which are mainly co-digested to enhance the digestion process and thus increase biogas yields [14]. According to the current efforts of developing a bio-based circular economy (German National Bioeconomy Strategy, https://biooekonomie.de/nationale-biooekonomiestrategie, accessed on 5 July 2021), there will be a transition to a residue-based biogas production. This is, however, associated with high demands on the system technology and, above all, the control of the complex, microbial-mediated process [3].

The anaerobic conversion of organic matter requires the collaboration of hundreds to thousands of microbial taxa of which Bacteria and Archaea have been most intensively studied [15,16,17,18,19]. Currently, taxonomic profiling of microbial communities is mostly achieved by 16S rRNA gene amplicon sequencing (e.g., [20]), whereby most studies only considered a limited number of anaerobic digesters. Previously, the microbial communities of 21 full-scale biogas plants were taxonomically profiled whereby the community composition was mainly affected by the process temperature and the type of the supplied feedstocks [21]. Likewise, in a more recent study which investigated the microbial community structures of 20 different full-scale anaerobic digestion plants over one year, the respective compositional patterns were related to the supplied feedstocks in terms of agricultural residues, bio-waste, municipal solid waste and sewage sludge [15]. In addition to the temperature and the supplied feedstocks, also the ammonium/ammonia concentration plays an important role in shaping the microbial community structure [22,23].

Despite intensive research, most of the microorganisms that are involved in the anaerobic digestion process, their activity under specific process conditions, as well as their response to changing environmental parameters are still unknown (e.g., [15,16,24]). In this regard, some studies revealed that changes in the microbial community compositions are rather reflected in the variability of rarely existing taxa and not by changes in the relative abundances of members of the so-called core microbiome [15,25,26,27]. However, the question arises, what are specific taxa for prevalent process conditions. Moreover, several relationships of different microbial taxa have already been identified within anaerobic digestion systems, indicating the occurrence of similar niche adaptations and close collaborations (e.g., [24,28,29,30]) while the overall interrelationships of the microbial taxa are still less understood. For example, there is evidence that members of the bacterial phyla Cloacimonetes and Bacteroidetes combined with the archaeal genus *Methanosaeta* are supposed to be indicative taxa for well-running mesophilic biogas processes due to their sensitive response to increasing ammonium nitrogen and volatile fatty acid concentrations, but knowledge is still lacking on the microbial interconnectivity, e.g., regarding a functional compensation by other microbiome members [25,29,31]. The phylum Actinobacteria is another interesting example. Members of this phylum are well-known as human pathogens and drug producers [32,33], but their role in anaerobic digestion systems is still unclear, although they have been found in high abundances [16], particularly in manure-based small biogas plants [29]. Last but not least, members of the archaeal phylum Bathyarchaeota were also recently recorded within anaerobic digestion systems [34], whereby their system ecological role is nearly unclear as members of this phylum have a wide range of metabolic capacities, including hydrolysis, acetogenesis, methane metabolism, dissimilatory nitrogen and sulfur reduction as well as interactions with acetoclastic methanogens or heterotrophic bacteria [35]. Thus, elucidating the biogas microbiome, as described by the taxonomical, functional and ecological diversity, is still required and of great value in order to optimize management measures by means of sufficient methane yields, to derive benchmarks that indicate impending critical process conditions and in particular to accompany the transition to a residue-based biogas production by providing recommendations for sustainable biogas plant operations based on resilient microbiomes.

The objective of this study was to investigate the microbial diversity of a representative number of German biogas plants with stable process conditions which differ in terms of plant configuration, supplied feedstocks, process temperatures, and thus in the prevalent chemical parameters. We hypothesize that (i) the investigated microbial communities vary in their structural composition according to the prevalent environmental conditions whereby marker microbiome cluster can be derived for specific process conditions, (ii) the marker microbiome clusters are characterized by indicative taxa, and (iii) these indicative taxa can be used to deduce/assess the general process conditions of the corresponding biogas digesters.

## 2. Materials and Methods

### 2.1. Sample Sources, Sample Collection and Chemical Analyses

In this study the microbial diversity of 67 anaerobic digesters belonging to 49 different agricultural biogas plants was investigated. These biogas plants constitute a representative number of the biogas plants operated in Germany which were monitored over one year as part of the “Third National Biogas Measuring Programm”. The selection of the biogas plants was based in particular on the following criteria: (i) Different feedstock compositions, e.g., plants which solely converted plant biomasses or animal manure as well as co-fermentation biogas plants, (ii) different plant technologies, e.g., fermenter designs, and (iii) different process operation strategies based on, e.g., process temperature, organic loading rates and retention times.

Sampling points for the microbiological inventory were chosen after the biogas plants have shown stable process conditions for at least three months. Samples of the digester content were taken from the main digesters, reflecting the digester content at the current operational conditions [31,36]. To reduce the microbial activity, taken samples were stored on ice and directly transferred to the laboratory where aliquots were taken and stored at −20 ${}^{\circ }$C until subsequent chemical and microbiological analyses. The biogas plant operators provided information concerning the prevalent process parameters and, if available, the produced biogas amount and composition. For the entire sample set operational parameters were available, which include the reactor types and volumes, the supplied feedstocks (varying mixtures of energy crops and residues from livestock husbandry), organic loading rate (OLR), hydraulic retention time (HRT) and process temperature (Table S1). To compare the feedstock inputs, the absolute mass-based values were normalized to 100% for each digester. For all samples the following chemical analyses were carried out: Total solids (TS), volatile solids (VS), pH value, total ammonium nitrogen (TAN), and volatile fatty acids (VFA) according to Liebetrau et al. [37]. The free ammonia (NH${}_{3}$) concentration was calculated as a function of the TAN concentration, the pH value and the temperature, according to Hansen et al. [38].

### 2.2. Taxonomic Profiling by Means of 16S rRNA Gene Amplicon Sequencing

The taxonomic profiling of the 67 biogas microbiomes was done as already described by Theuerl et al. [29]. Briefly, DNA was extracted from three replicates per sample using the FastDNA^®^ Spin Kit for Soil (MP Biomedicals, France) in combination with the Genomic DNA Clean & Concentrator${}^{\mathrm{TM}}$ kit (Zymo Research, Irvine, CA, USA). The 16S rRNA gene amplicon library preparation was done applying the “16S Metagenomic Sequencing Library Preparation” protocol (Illumina Inc., San Diego, CA, USA) while using the universal primer pair Pro341F (5′-CCTACGGGNBGCASCAG-3′) and Pro805R (5′-GACTACNVGGGTATCTAATCC-3′) for amplification of the V3–V4 hypervariable regions [39]. The amplicon libraries were sequenced on the Illumina MiSeq platform applying the paired-end protocol for 300 bp reads and afterwards demultiplexed. The bioinformatic processing of the 16S rRNA gene amplicon sequences started with a quality control of the raw sequencing reads with FastQC. Afterwards the forward and reverse reads were merged with FLASH [40] and the primers were removed with cutadapt [41]. Sickle was used for trimming by quality of the merged reads [42]. The high-quality reads of each sample were subsampled to a given depth (50,000 reads) using seqtk (https://github.com/lh3/seqtk, accessed on 5 July 2021). Within the QIIME platform [43] usearch61 was used, for de novo (abundance based) and reference-based chimera detection. After this step, an open reference based operational taxonomic unit (OTU) clustering and a taxonomic assignment was accomplished while using the 16S rDNA SILVA database (release 132) as reference. Finally, triplicates of the biogas digesters were used to calculate median abundance values which were afterwards normalized to 100%.

### 2.3. Statistical Data Analysis

In order to evaluate correlations among and between biotic and abiotic system parameters, non-metric multidimensional scaling (NMDS) [44,45,46] was performed with the R Project for Statistical Computing [47] using the package “vegan” [48] and “gplots” [49]. The distance matrix was calculated using the McQuitty algorithm [50]. The calculated (dis)similarities were first visualized as a tree diagram to define the existing clusters of the investigated anaerobic digesters based on their taxonomic profiles. Subsequently, the calculated distance matrix was used to visualize colored NMDS ordination plots which were combined with environmental vectors based on the process parameters calculated by the function “envfit” [48]. The results were sorted according to the *R${}^{2}$* values while only vectors with *R${}^{2}$* > 0.3 and *p* < 0.001 were considered.

To identify taxa which are indicative for specific environmental conditions, an indicator species analysis (ISA) according to [51] was performed using the “indicspecies” package of R with a point biserial correlation coefficient as statistic value [52,53]. Considering only indicative taxa with a point biserial correlation coefficient above 0.5 and *p*-values below 0.0005.

Pearson correlations between the process parameters and the indicator species abundances were calculated in R using the “cor” function of the package “stats” [47]. The results were sorted according to the correlation values, while moderate correlations between ±0.50 and ±0.75 and strong correlations higher/lower than ±0.75 were considered.

## 3. Results and Discussion

### 3.1. Operational and Chemical Characteristics of the 67 Analyzed Biogas Digesters

**Figure 1:**
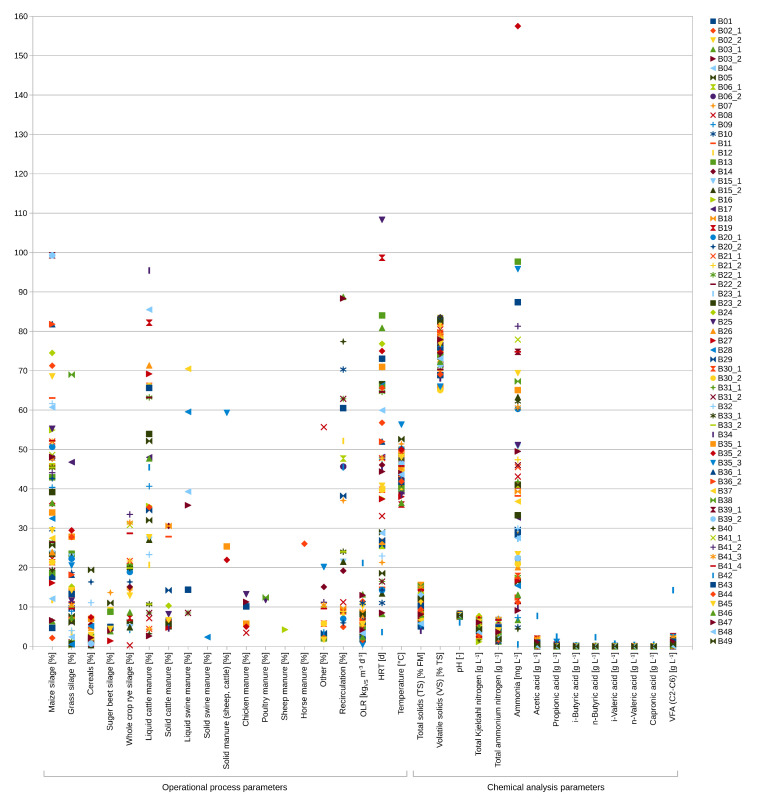
Range of the operational and chemical process parameters from the 67 analyzed anaerobic digesters. The different units of the paramaters are indicated on the *x*-axis. Supplied feedstocks were normalized to 100% for each digester. The acid concentrations were summarized as volatile fatty acids (VFA) given as acetic acid equivalents. OLR = organic loading rate, HRT = hydraulic retention time.

The investigated 67 anaerobic digesters belonging to 49 agricultural full-scale biogas plants differed in their prevalent operational process conditions (Figure 1, Table S1). The main feedstocks were maize and grass silage (in 60 and 48 digesters) as well as liquid and solid cattle manure (in 36 and 20 digesters) which ranged from 2.1 to 99.3%, 0.3 to 69.0%, 2.6 to 95.5% and 4.6 to 30.6% of the total feedstock supply, respectively. Further rarely or occasionally used feedstocks were sugar beet silage in ten digesters (1.4–13.7%), whole crop rye silage in 24 digesters (0.3–33.5%), cereals in 28 digesters (0.3–19.4%), liquid and solid swine manure in eight digesters (8.5–70.5%), chicken/poultry manure in eight digesters (3.4–25.0%), sheep manure in one digester (4.2%) and horse manure in one digester (26.1%). The relative shares of the used feedstocks are in good agreement with the average feedstock compositions of German biogas plants [13].

The OLRs of the biogas digesters ranged from 0.4 to 21.2 kg${}_{\mathrm{VS}}$m${}^{-3}$d${}^{-1}$ with corresponding HRTs from 3.6 to 475.0 days. The process temperature varied between 36.0 ${}^{\circ }$C and 56.3 ${}^{\circ }$C, whereby 57 of the 67 analyzed anaerobic digesters were operated at a process temperature between 39 ${}^{\circ }$C and 49 ${}^{\circ }$C, a range between the optimal condition for mesophilic (33–38 ${}^{\circ }$C) or thermophilic (50–60 ${}^{\circ }$C) process operation, respectively [23,54]. Three biogas plants were operated at mesophilic conditions (≤38 ${}^{\circ }$C) and seven biogas plants at thermophilic conditions (≥50 ${}^{\circ }$C). Due to the broad variety of the operational process parameters, differences in the chemical process parameters were expected. Hence, the pH values ranged from 6.1 to 8.3, the TKN concentrations from 2.6 to 7.7 gL${}^{-1}$, the TAN concentrations from 1.2 to 4.7 L${}^{-1}$, the NH${}_{3}$ concentrations from 0.5 to 157.5 mgL${}^{-1}$ and the VFA concentrations from below the detection limit up to 14.3 gL${}^{-1}$ (Figure 1, Table S2).

### 3.2. Taxonomic Profiling of the 67 Analyzed Anaerobic Digesters

**Figure 2:**
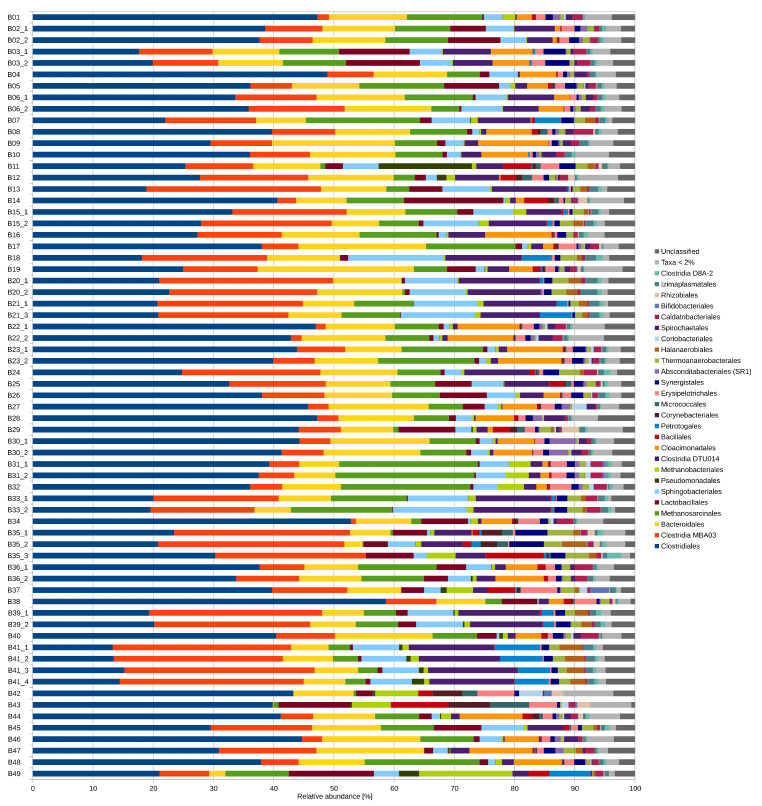
Taxonomic profiles on order level of the 67 analyzed anaerobic digesters from 49 agricultural, full-scale biogas plants as deduced from 16S rRNA gene amplicon data. Orders with a maximal relative abundance less than 1% were summarized. Taxa which were not taxonomically assigned at order level were summarized as “unclassified”.

This study investigated the taxonomic diversity of 67 anaerobic digesters originating from 49 different agricultural full-scale biogas plants. The taxonomic profiles of the biogas microbiomes were determined by 16S rRNA gene amplicon sequencing. A total of 17,556 different OTUs with an average number of 2260 ± 528 (Table S3) were identified per digester. The taxonomic profiles revealed that 89.4% ± 6.5% of the detected taxa were assigned to the domain Bacteria, 9.4% ± 6.3% to the domain Archaea and 1.2% ± 0.6% to taxonomically unclassified organisms. The dominant bacterial phyla were Firmicutes (60.4% ± 8.2%), Bacteroidetes (15.3% ± 5.1%), Cloacimonetes (3.2% ± 3.7%), Actinobacteria (1.6% ± 3.0%), Tenericutes (1.4% ± 0.6%) and Atribacteria (1.2% ± 0.8%) (Figure S1). On the order level, Clostridiales were dominantly found in all analyzed digesters with 32.5% ± 10.8%, followed by “uncultured Clostridia MBA03” with 13.4% ± 9.1%, Bacteroidales with 10.8% ± 4.4%, “uncultured Clostridia DTU014” with 5.3% ± 4.6%, Sphingobacteriales with 4.4% ± 3.4%, Lactobacillales with 3.4% ± 3.8% and Cloacimonadales with 3.2% ± 3.7% (Figure 2). Among the domain Archaea, members of the phylum Euryarcheota were predominantly found in all analyzed anaerobic digesters with 9.3% ± 6.3%. Other archaeal phyla, such as Crenarchaeota, Nanoarchaeota and Diapherotrites, were found with low relative abundances of maximal 0.2%, 0.1% and 0.03%, respectively. On the order level, Methanosarcinales dominated the analyzed digesters with 7.9% ± 5.8%, followed by Methanobacteriales with 1.3% ± 2.3% (Figure 2). The recorded taxonomic profiles, in general, correspond to the expected taxonomic composition of microbial communities in biogas digesters [15,21,22,55].

### 3.3. Marker Microbiome Clusters Depending on Prevalent Process Conditions

**Figure 3:**
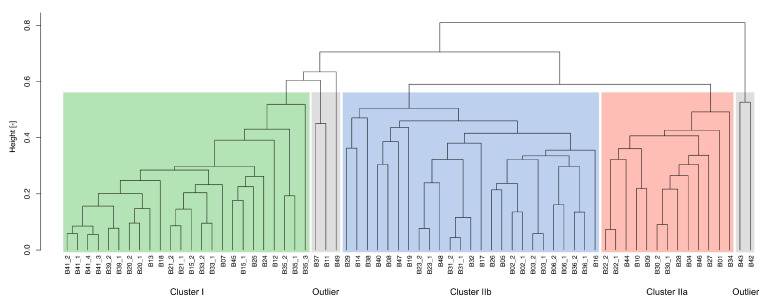
Tree diagram based on a McQuitty (dis)similarities matrix calculated from the taxonomic profiles of the analyzed 67 anaerobic digesters from 49 agricultural biogas plants.

In order to identify potential marker microbiome clusters for prevalent process conditions, different statistical analyses were carried out. In a first step, a McQuitty (dis)similarity matrix was calculated and visualized in a tree diagram using the taxonomic profiles of the 67 investigated anaerobic digesters (Figure 3). Due to the taxonomic diversity of the analyzed anaerobic digesters, they were divided into two main clusters I and II and two groups of outliers. Cluster I represents digesters featuring process temperatures higher than 45 ${}^{\circ }$C, whereas cluster II comprises digesters with process temperatures lower than 45 ${}^{\circ }$C. Cluster II was further separated into two distinct subclusters IIa and IIb (Figure 3).

Subsequently, the defined microbiome clusters were used to visualize colored NMDS ordination plots which arrange objects, in this case the recorded taxonomic profiles, as close as possible according to their (dis)similarities. The identified clusters were coloured according to the defined clusters of the tree diagram: Cluster I in green, cluster IIa in red, cluster IIb in blue and the outliers in grey (Figure S2). Many of the investigated samples were assigned to one of the specific microbiome clusters I, IIa and IIb based on the calculated McQuitty (dis)similarity matrix visualized as tree diagram (Figure 3). However, the NMDS ordination plot revealed, in addition to the outliers of the tree diagram (B11, B37, B42, B43, B49), samples which also lie outside of the identified clusters (B12, B14, B19, B25, B29, B35, B38, B44) (Figure S2). These samples will be summarized as group of outliers for the following analyses.

**Figure 4:**
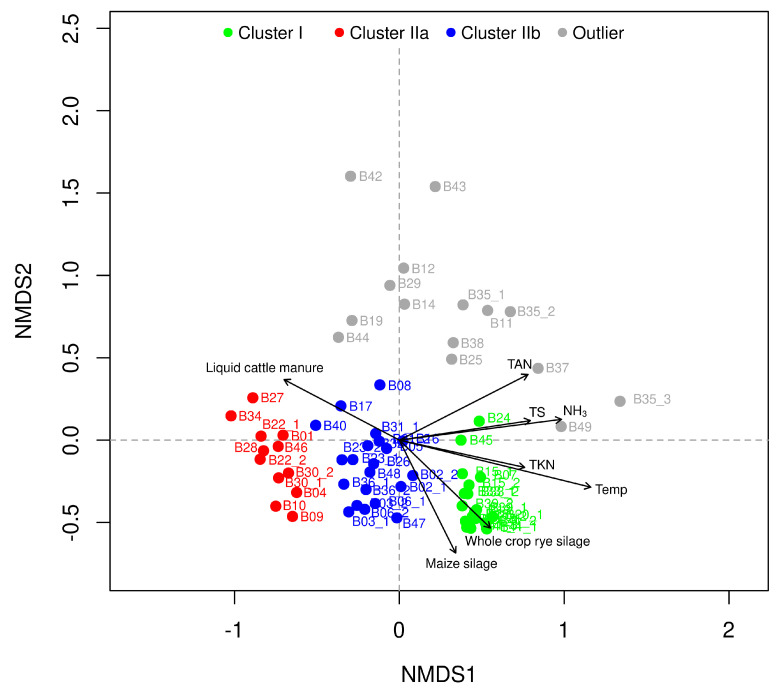
Non-metric multidimensional scaling (NMDS) based on the taxonomic diversity of the 67 analyzed digester samples from 49 agricultural, full-scale biogas plants. The color code is deduced from the tree diagram in Figure 3: Cluster I = green, cluster IIa = red, cluster IIb = blue, outlier = gray. The given environmental vectors symbolize the amount of maize silage (*R${}^{2}$* = 0.30, *p* = 0.001), whole crop rye silage (*R${}^{2}$* = 0.30, *p* = 0.001), liquid cattle manure (*R${}^{2}$* = 0.32, *p* = 0.001), the process temperature (*R${}^{2}$* = 0.74, *p* = 0.001), total solids (TS) (*R${}^{2}$* = 0.33, *p* = 0.001), the total ammonium nitrogen (TAN) (*R${}^{2}$* = 0.40, *p* = 0.001), the total Kjeldahl nitrogen (TKN) (*R${}^{2}$* = 0.31, *p* = 0.001) and the free ammonia nitrogen concentration (NH${}_{3}$) (*R${}^{2}$* = 0.51, *p* = 0.001).

As expected, the NMDS analysis revealed cluster I (green in Figure 3 and Figure 4) for which the process temperature had a significant impact (*R${}^{2}$* = 0.74, *p* = 0.001) on the formation of the microbial community structure. This cluster consists of 19 anaerobic digesters from 11 biogas plants (B07, B13, B15, B18, B20, B21, B24, B33, B39, B41, B45); all of them showed a process temperature higher than 45 ${}^{\circ }$C (Table S1). The separation of this cluster from the other anaerobic digesters is in accordance with previously published studies which identified the process temperature as one of the main factors for the shaping of microbial communities within anaerobic digesters [22,28,31,56,57].

Among the available operational and chemical process parameters, the process temperature had the strongest impact on separating the two main microbiome clusters I and II (Figure 3). Due to their lower correlation values, all other process parameters can only to a certain degree be used to determine the influence of the abiotic environment on the taxonomic diversity. Considering a correlation tendency, also the amount of liquid cattle manure (*R${}^{2}$* = 0.32, *p* = 0.001), rye and maize silage (each with a *R${}^{2}$* of 0.30, *p* = 0.001), the TAN concentration (*R${}^{2}$* = 0.40, *p* = 0.001), the TKN concentration (*R${}^{2}$* = 0.31, *p* = 0.001) as well as the NH${}_{3}$ concentration (*R${}^{2}$* = 0.51, *p* = 0.001), might explain the taxonomic composition of the clusters (Figure 4). Most probably these low correlations are related to the chemical similarities of the used feedstocks in terms of energy crops and residues from livestock husbandry in combination with an almost similar process operation (Table S1). This is in accordance with previous studies which have shown stronger significant correlations of the microbial community structure with prevalent process parameters, e.g., the supplied feedstocks. Hence, differentiations occur when agricultural biogas plants are compared with anaerobic digestion plants using either chemically complex and/or heterogeneous feedstocks in function of time, (e.g., biowaste which is the most variable feedstock as its chemical composition, for example, is subjected to seasonal differences) or more homogeneous feedstocks (e.g., wastewater sludge and industrial waste) [15,22,24,28,31]. Independently of these similarities within the process parameters of the investigated biogas plants, the question arises whether indicative microbiome clusters can be derived for specific process conditions. In this regard, the NMDS analysis revealed the two subclusters, IIa and IIb, of biogas plants operated at a process temperature lower than 45 ${}^{\circ }$C. The subcluster Ia (marked in red in Figure 3 and Figure 4) consists of 12 anaerobic digesters from ten biogas plants (B01, B04, B09, B10, B22, B27, B28, B30, B34, B46) which were operated with a balanced mixture of energy crops (mainly maize and grass silage and occasional addition of sugar beet silage or cereals) and residues from livestock husbandry (liquid and solid cattle and swine manure) with a process temperature between 36 ${}^{\circ }$C and 43 ${}^{\circ }$C (Table S1). These plants showed none of the commonly known critical features in their chemical parameters (Table S2) such as elevated TAN or VFA concentrations [19,58,59]. Compared to this, subcluster IIb (marked in blue in Figure 3 and Figure 4) consists of 21 anaerobic digesters from 15 biogas plants (B02, B03, B05, B06, B08, B16, B17, B23, B26, B31, B32, B36, B40, B47, B48) which were operated at similar feedstock and temperature conditions as the digesters from cluster IIa, but differ by slightly higher values of the pH (7.2 ± 0.2 vs. 7.8 ± 0.7), the VFA concentration (0.07 ± 0.07 gL${}^{-1}$ vs. 0.54 ± 0.70 gL${}^{-1}$) and the TAN concentration (1.8 ± 0.4 gL${}^{-1}$ vs. 2.2 ± 0.7 gL${}^{-1}$) (Table S2).

For the previously identified group of outliers, other environmental factors than the prevalent process temperature or slight variations of certain chemical parameters led to a different composition of the corresponding microbial communities. With respect to the supplied feedstocks, it becomes obvious that the corresponding microbiomes are either affected by comparatively high amounts of solid and liquid cattle manure (samples B12, B14, B19, B29, B42, B43, B44) or by relatively high amounts of chicken and poultry manure resulting in elevated pH values as well as TAN and NH${}_{3}$ concentrations (B11, B25, B38, B35, B37, B49) (Figure 4, Tables S1 and S2).

In accordance with these results, former cluster (PCoA, NMDS) and co-occurrence network analyses indicate the existence of specific microbial assemblages for different anaerobic digestion systems [15,22,28,31] while it has to be considered that these studies compared agricultural biogas plants with anaerobic digestion systems converting either biowaste or wastewater which significantly differ. In contrast, this study compared agricultural biogas plants that are more similar regarding their input feedstocks and process parameters (see Section 3.1). At this point, the question arises whether there are indicative taxa which are responsible for the identified clustering.

### 3.4. Indicative Taxa for Prevalent Process Conditions

In order to find indicative taxa for the three identified clusters I, IIa, and IIb as well as for the group of outliers, an Indicator Species Analysis (ISA) was calculated based on the taxonomic profiles of all digester samples. Considering only indicative taxa with a point biserial correlation coefficient above 0.5 and *p*-values below 0.0005, overall 253 taxa were identified as differentially abundant between the three defined clusters and cluster combinations: 23 indicative taxa were identified for cluster I, 68 taxa for cluster IIa, and four taxa for cluster IIb (Table S4). For the outlier group, 123 taxa were identified as potentially indicative, which have to be handled carefully due to the broad dispersion of the outlier samples. The remaining 35 recorded indicative taxa refer to combinations of two or three clusters, whereby 28 of them are indicative for the combination of cluster IIa and IIb.

**Figure 5:**
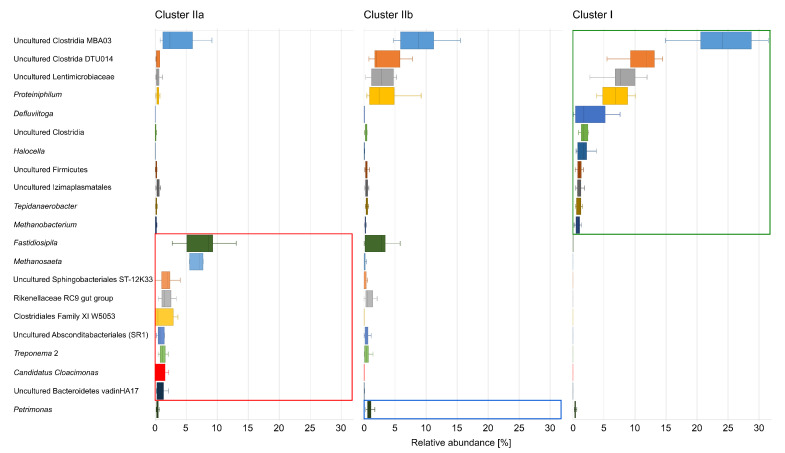
Exemplary distribution of the 21 most abundant indicative taxa and their relative abundances within the defined clusters IIa, IIb, I based on the taxonomic profiles. Indicator taxa of the respective clusters are boxed in red for cluster IIa, blue for cluster IIb and green for cluster I. Statistically significant differences were calculated using the “indicspecies” package of R with a point biserial correlation coefficient as statistic value [52,53].

The microbial composition of cluster I, which consists of anaerobic digesters with elevated process temperatures between 45 ${}^{\circ }$C and 56 ${}^{\circ }$C, is mainly characterized by members of uncultured genera of the class Clostridia (Figure 5, Table S4). In particular, two groups, namely “uncultured Clostridia MBA03” and “uncultured Clostridia DTU014”, showed 24.0% ± 4.9% and 11.2% ± 2.5%, respectively, the highest relative abundances of the indicator taxa. Further indicator taxa with high abundances were assigned to the genera “uncultured Lentimicrobiaceae” with 8.4% ± 2.7%, *Proteiniphilum* with 6.9% ± 2.0%, *Defluviitoga* with 2.6% ± 2.5%, “uncultured Clostridia” with 1.8% ± 0.5% and *Halocella* with 1.7% ± 0.9%. The archaeal community was solely represented by the genus *Methanobacterium* with a relative abundance of 0.9% ± 0.4% (Figure 5, Table S4). These findings are in general accordance with previously published studies which highlighted the process temperature as one of the main factors for shaping of the microbial community structure within anaerobic digesters. These microbial communities are predominated by members of the phylum Firmicutes, especially of the class Clostridia and the phylum Thermotogae as well as hydrogenotrophic methanogens such as the archaeal genus *Methanobacterium* [22,24,28,31,56,60]. Surprisingly, members from the phylum Bacteroidetes by means of the genera “uncultured Lentimmicrobiaceae” and *Proteiniphilum* were identified as indicative taxa for cluster I. It is commonly reported that the relative abundance of members of the phylum Bacteroidetes is significantly lowered at thermophilic process conditions [22,23,28,61] which is supported as the known and described species of these both genera have their optimal growth conditions at 30–37 ${}^{\circ }$C (*Lentimicrobium saccharophilum*, [62]), at 37 ${}^{\circ }$C (*Proteiniphilum acetatigenes*, [63]) or at 35–40 ${}^{\circ }$C (*Proteiniphilum saccharofermentans*, [64]). However, as a reliable assignment of the detected sequences is only possible at the genus level [20,65,66], both mentioned genera probably comprise thermophilic or at least thermo-tolerant species.

However, Pearson correlations of the indicative taxa with the process parameters showed moderate positive correlations (between 0.50 and 0.75) with the amount of whole crop rye silage and/or the NH${}_{3}$ concentration in particular for the bacterial genera “uncultured Clostridia MBA03”, “uncultured Clostridia DTU014”, “uncultured Lentimicrobiaceae”, *Halocella* and the archaeal genus *Methanobacterium* (Table S6). Taking into further consideration that the bacterial genera “uncultured Clostridia MBA03” and “uncultured Clostridia DTU014” showed a moderate negative correlation (−0.54 and −0.52, respectively) with the feedstock liquid cattle manure (Table S6), it might be assumed that the members of these both genera are involved in the degradation of plant biomass. This strengthens their importance of being indicative for the process condition of the anaerobic digestion systems they are living in.

Cluster IIa consists of biogas plants characterized by very low values of the prevalent chemical process parameters (Table S2). The ISA revealed that most of the 68 recognized indicative taxa for this cluster belong to the bacterial phyla Bacteroidetes (16.4%), Firmicutes (16.4%) and Patescibacteria (11.9%), followed by the phyla Spirochaetes (9%), Chloroflexi (9%), Planctomycetes (4.5%) as well as Atribacteria, Cloacimonetes, Kiritimatiellaeota and Synergistetes (each 3%) (Figure 5, Table S4). This picture is also supported considering the 22 most abundant genera: Six belong to the phylum Firmicutes (with relative abundances between 0.2% ± 0.1% and 7.6% ± 2.8%), five to the phylum Bacteroidetes (with relative abundances between 0.6% ± 1.2% and 1.9% ± 1.1%), and two genera belong to each of the phyla Patescibacteria, Spirochaetes and Chloroflexi (with relative abundances between 0.2% ± 0.2% and 1.3% ± 1.2%) (Table S4). With one genus each, the bacterial phyla Cloacimonetes (*Candidatus Cloacimonas*, 1.0% ± 0.8%), and Synergistetes (“uncultured Synergistaceae”, 0.5% ± 0.1%) also belong to the most abundant indicative taxa of cluster IIa. For the archaeal community, the genus *Methanosaeta* was identified as an indicative taxon with a relative abundance of 6.3% ± 2.9% (Figure 5, Table S4). This is partly in accordance with previously published results by Theuerl et al. [29,31], where the occurrence of members from the bacterial phyla Bacteroidetes and Cloacimonetes and the archaeal genus *Methanosaeta* (syn. *Methanothrix*) were related to unstressed anaerobic digestion systems with low concentrations of potential process inhibitory factors. In comparison, this study showed that much more members of various phyla are potentially indicative for unstressed process conditions, besides the already known taxa. In particular, these include members of the phyla Firmicutes, Patescibacteria, Spirochaetes and Chloroflexi.

However, out of the 68 indicative taxa for cluster IIa, for ten taxa belonging to the phyla Bacteroidetes, Firmicutes, Patescibacteria, Spirochaetes, Atribacteria and Kiritimatiellaeota positive correlations (from 0.51 to 0.79) were detected, in particular for the process parameters liquid cattle manure and swine manure (Table S6). In addition, two indicative taxa assigned inter alia to the nine most abundant indicative taxa showed moderate negative correlations with the NH${}_{3}$ concentration (Table S6) which supports the assumption that the identified taxa are indicative for mesophilic unstressed anaerobic digestion systems with low concentrations of potential process inhibitory factors, here in terms of the NH${}_{3}$ concentration.

Cluster IIb includes digester samples from co-fermentation plants, which differ from cluster IIa by slightly elevated pH values, VFA and TAN concentrations (Table S2). The corresponding microbial communities were predominantly characterized by the indicative genus *Petrimonas* (0.9% ± 0.4%) of the phylum Bacteroidetes (Table S4). The remaining three indicative genera “uncultured Chthonomonadales”, *Ruminiclostridium* 6 and “uncultured BRC1” showed relative abundances below 0.01%.

Since the process conditions of cluster IIa and IIb are very similar and only differ slightly in values for the parameters pH, TAN and VFA, it was expected that a large group of indicative taxa were identified for the combination of these two clusters vs. cluster I. Out of the 28 indicative taxa for cluster IIa and IIb, 43% belong to the phylum Firmicutes, 29% to the phylum Bacteroidetes and 7% to the phylum Cloacimonetes. The phyla Actinobacteria, Chloroflexi, Fibrobacteres, Synergistetes, Tenericutes and Verrucomicrobia are each represented by one genus among the identified indicative taxa. The most abundant genera were *Sedimentibacter* (Firmicutes, 6.6% ± 2.3%), “uncultured Ruminococcaceae” (Firmicutes, 2.0% ± 0.5%), *Herbinix* (Firmicutes, 1.6% ± 0.6%), “uncultured Bacteroidales UCG-001” (Bacteroidetes, 2.1% ± 1.4%), *Fermentimonas* (Bacteroidetes, 0.9% ± 0.4%) and “uncultured Cloacimonadaceae W5” (Cloacimonetes, 3.3% ± 2.8%) (Table S4).

Compared to cluster I, correlations of the indicative taxa of cluster II (a and b) with the process parameters showed an opposite behavior by means of negative correlations with the NH${}_{3}$ concentration. Hereby, 17 indicative taxa showed moderate negative correlation values between −0.50 and −0.62 for the NH${}_{3}$ concentration (Table S6), indicating that these taxa from cluster II are potentially sensitive to elevated NH${}_{3}$ conditions.

For the group of outliers, 123 indicator taxa were identified. These taxa have to be considered carefully due to the broad dispersion of the outlier samples. However, 40% of these taxa were assigned to the phylum Actinobacteria and 30% to the phylum Proteobacteria (Table S4). In no other cluster Proteobacteria were identified as indicator taxa and only four genera of the phylum Actinobacteria were identified in cluster II. Additionally, approximately 50% of the indicative taxa assigned to the phylum Actinobacteria showed moderate positive correlations (from 0.50 to 0.70) with the parameters solid cattle manure, chicken manure, poultry manure as well as horse manure (Table S6). Thus, it can be assumed that taxa of these phyla seem to be indicators for biogas digesters with higher amounts of animal manure. Regarding the high occurrence of members from the phylum Actinobacteria, this assumption is supported by several comparative studies including anaerobic digestion systems with higher shares of excrements (manure from livestock husbandry or wastewater sludge) [15,22,29,55,67]. However, studies considering the microbial community structure of anaerobic digestion systems compared with the gastrointestinal tract of farm animals as well as the corresponding manures, in order to elucidate the system specificity of Actinobacteria and especially Proteobacteria and their origin, are still lacking.

In conclusion, the taxonomic profiling enabled the definition of three biogas microbiome clusters. Within these clusters, taxa and their specific abundance ranges were identified which are indicative for the corresponding type of process condition.

### 3.5. Clustering of the Biogas Microbiomes by Indicative Taxa and Generally Occurring Taxa

**Figure 6:**
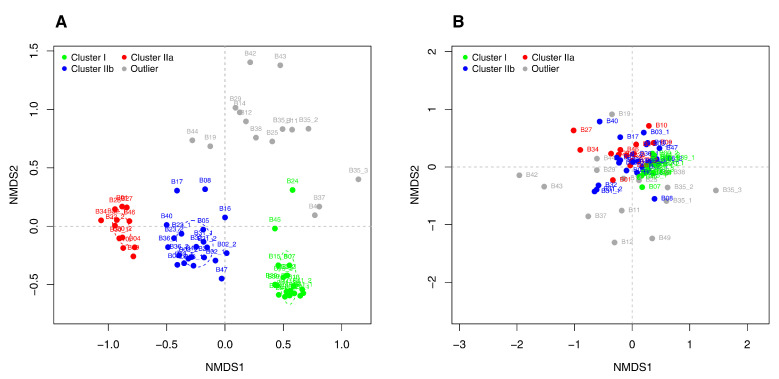
Non-metric multidimensional scaling (NMDS) (**A**) based on the identified indicative taxa as deduced from indicator species analyses (ISA) and (**B**) the generally occurring taxa of the 67 analyzed digesters from 49 agricultural, full-scale biogas plants. The color code corresponds to the clusters in Figure 4: I = green, IIa = red, IIb = blue, outlier = gray.

In order to verify the identified indicative taxa as deduced from ISA for their usability to assess the identified clusters based on the entire taxonomic microbial diversity, further NMDS analyses were calculated (Figure 6A). These analyses revealed that it is possible to distinguish the same three main clusters and the group of outliers which could be distinctly separated from each other by just using the 253 indicative genera and their relative abundances instead of 1021 genera from the entire microbial community. Besides the mentioned indicative taxa (see Section 3.4), also 415 generally occurring genera were observed within the taxonomic profiles of the analysed digesters. They mainly show similar relative abundances within the three clusters and the group of outliers, or between cluster combinations. The most abundant (>1%) generally occurring genera were *Ruminiclostridium*, *Clostridium sensu stricto* 1, “uncultured Syntrophomonadaceae” and *Ruminiclostridium* 1 (Table S5). Further generally occurring taxa with partly quite different average relative abundances within the clusters were “uncultured Rikenellaceae” (cluster I: <0.1%, cluster IIa: 1.5%, cluster IIb: 0.7%, outlier: 0.9%), *Methanobrevibacter* (cluster I: <0.1%, cluster IIa: 0.4%, cluster IIb: 0.7%, outlier: 1.3%) and *Pseudomonas* (cluster I: 0.2%, cluster IIa: <0.1%, cluster IIb: <0.1%, outlier: 1.2%). These taxa were not assigned as indicator taxa, which could be explained by abundance variations in the respective clusters. As expected, it could be shown that the generally occurring taxa do not enable any clustering (Figure 6B). This indicates that certain process conditions are not reflected by members of the core-microbiome, but rather by specific taxa, which are adapted to the prevailing conditions as it was shown in wastewater treatment systems or anaerobic digestion plants (e.g., [15,27,68]).

However, from an ecological point of view according to the Pareto principle, generalists which exist in high and even abundances under various conditions are supposed to be responsible for 80% of the energy flux and hence they are important drivers for the overall process functioning [15,31,69]. Consequently the specialists, by means of the identified indicative taxa, of the biogas microbiomes occupy specific ecological niches and hence can be used to assess certain process conditions more precisely than the whole microbiome or the usually used process parameters. It is hypothesized that the reaction of the microbial communities, especially the indicative taxa, enables an earlier response to changing environmental conditions, than the measurable chemical process parameters show elevated or critical values. Thus process specific indicative taxa might be used as sensitive markers for an early insight into the process condition of agricultural biogas plants in the future.

## 4. Conclusions

In this study, 67 microbiomes originating from 49 agricultural full-scale biogas plants converting energy crops and/or residues from livestock husbandry were taxonomically investigated. As it was hypothesized, the investigated microbial communities vary in their structural composition according to specific process conditions whereby three marker microbiome clusters, I, IIa and IIb, and one group of outliers were identified. For the first time, this study has shown that these marker microbiome clusters are characterized by specific genera, here by 253 indicative taxa which are differentially abundant within the defined clusters. Correlation analyses of the 253 indicative taxa against the prevailing process parameters supported and specified the occurrence of microbial indicators for certain process conditions, in particular the presence of potential indicators for mesophilic unstressed conditions as well as the presence of taxa that are adapted to higher NH${}_{3}$ concentrations with an overall adequate process stability. For the first time, this study showed the occurrence of potential sensitive and resistant/resilient taxa e.g., to elevated NH${}_{3}$ concentrations, which leads to the assumption that such indicative taxa provide better information of the actual process conditions than the entire microbiome (with more than 1000 different genera) or the usually used chemical process parameters such as the acid and/or the ammonium/ammonia concentration. However, at this point the question arises how these indicative taxa and hence the entire system will respond to changing environmental conditions, especially the transition to a residue-based biogas production and the related frequently varying process conditions, since they are obviously highly adapted to the prevalent process conditions of their anaerobic digestion system. To follow up, there is a need to find out, whether these indicative taxa have specific tolerance ranges to the prevailing environmental conditions in order to develop easy-to-use detection systems which information can be used for the development of advanced, data-driven process models that require benchmarks for both stable/resilient and impending critical process conditions in order to recommend sustainable biogas plant operations.

From a scientific point of view, insights into the metabolic potential of entire microbial communities from various anaerobic digestion systems of the here defined microbiome clusters through metagenomic analyses as well as of genetic specifications of the occurring, in particular yet unknown microorganisms through genome reconstruction approaches combined with cultivation approaches would enable a deeper understanding of the interconnections between process conditions and the composition of the microbial community with its functional potential. Additionally, the actually realized functions as deduced from metaproteomic analyses would enable the analysis of the metabolic solutions of cluster specific microbial communities to fulfill the anaerobic digestion chain in biogas plants.

Taking into account that the currently available methods for investigating the microbial diversity are highly complex in terms of sample preparation and, in particular, data evaluation and interpretation, identifying microbial indicators (at the taxonomic and functional level) for specific process conditions is a precondition for the development of rapid and economical microbial detection systems and hence new process models. These can be used by plant operators and consultants for monitoring, assessing and managing all types of anaerobic digestion systems using e.g., sewage sludge, residues from livestock husbandry, landscape management, from food processing and consumption as well as from biorefineries, or the organic fraction of municipal solid waste.

## Data Availability

The raw 16S rRNA gene amplicon sequences are deposited in the European Nucleotide Archive (ENA) under the study accession PRJEB39821.

## Supplementary Materials

The following are available online at https://www.mdpi.com/article/10.3390/microorganisms9071457/s1: Figure S1: Taxonomic profiles at phylum level for the 67 investigated biogas microbiomes from 49 agricultural, full-scale biogas plants. Phyla with a maximal relative abundance less than 1% were summarized. Taxa which were not taxonomically assigned at phylum level were summarized as “unclassified”. Figure S2: Non-metric multidimensional scaling (NMDS) based on the taxonomic diversity of the 67 analyzed digester samples from the 49 agricultural, full-scale biogas plants. The color code of the clusters is deduced from the tree diagram in Figure 3: I = green, IIa = red, IIb = blue, outliers = gray. The given environmental vectors symbolize the amount of maize silage (*R${}^{2}$* = 0.30, *p* = 0.001), whole crop rye silage (*R${}^{2}$* = 0.30, *p* = 0.001), liquid cattle manure (*R${}^{2}$* = 0.32, *p* = 0.001), the process temperature (*R${}^{2}$* = 0.74, *p* = 0.001), total solids (TS) (*R${}^{2}$* = 0.33, *p* = 0.001), the total ammonium nitrogen (TAN) (*R${}^{2}$* = 0.40, *p* = 0.001), the total Kjeldahl nitrogen (TKN) (*R${}^{2}$* = 0.31, *p* = 0.001) and the free ammonia nitrogen concentration (NH${}_{3}$) (*R${}^{2}$* = 0.51, *p* = 0.001). Figure S3: Taxonomic profiles at genus level for the 67 investigated biogas microbiomes from 49 agricultural, full-scale biogas plants sorted by their respective cluster. Genera with a maximal relative abundance <5.5% were summarized. Taxa which were not assigned at genus level were summarized as “unclassified”. Table S1: Overview of the operational parameters of the 67 analyzed biogas digesters from 49 agricultural, full-scale biogas plants. The used feedstocks were normalized to 100% for each biogas digester. Table S2: Overview of the chemical parameters of the 67 analyzed biogas digesters from 49 agricultural full-scale biogas plants. VFA = volatile fatty acids. Table S3: Overview of the detected operational taxonomic units (OTUs) as well as the calculated α-diversity indices in terms of Shannon, Simpson and Chao1 for the investigated 67 biogas digesters from 49 agricultural, full-scale biogas plants. Table S4: The 23 most abundant indicative taxa above a maximal relative abundance of 0.5%, their average abundances and standard deviations within the defined clusters based on the taxonomic profiles. Statistically significant differences were calculated using the “indicspecies” package of R with a point biserial correlation coefficient as statistic value [De Cáceres & Legendre, 2009; De Cáceres et al., 2011]. Table S5: The most abundant generally occurring taxa above a maximal relative abundance of 0.5%, their average abundances and standard deviations within the defined clusters based on the taxonomic profiles. Table S6: Correlations of 123 indicator taxa against 18 process parameters. Shown are only moderate correlations between ±0.50 and ±0.75 and strong correlations above/below ±0.75.
