# Supplementary figures and images for: Indicative Marker Microbiome Structures Deduced from the Taxonomic Inventory of 67 Full-Scale Anaerobic Digesters of 49 Agricultural Biogas Plants
Source: Microorganisms. 2021 Jul 7;9(7):1457. doi: 10.3390/microorganisms9071457 (PMC8307424; doi:10.3390/microorganisms9071457)

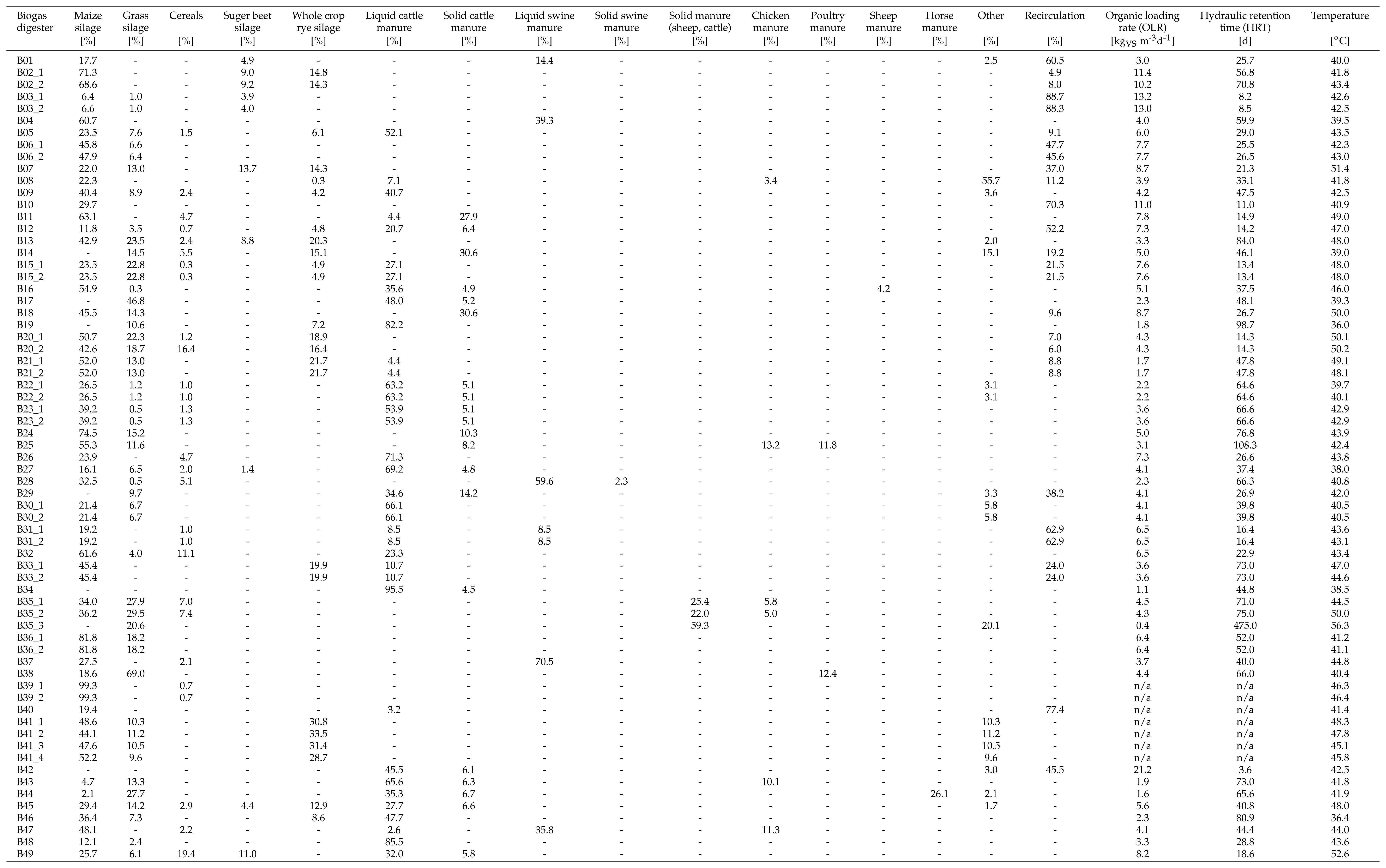

Supplement: Supplementary file 1 [file microorganisms-09-01457-s001.zip › Supplementary_Materials_Proof/Table_S1.png]

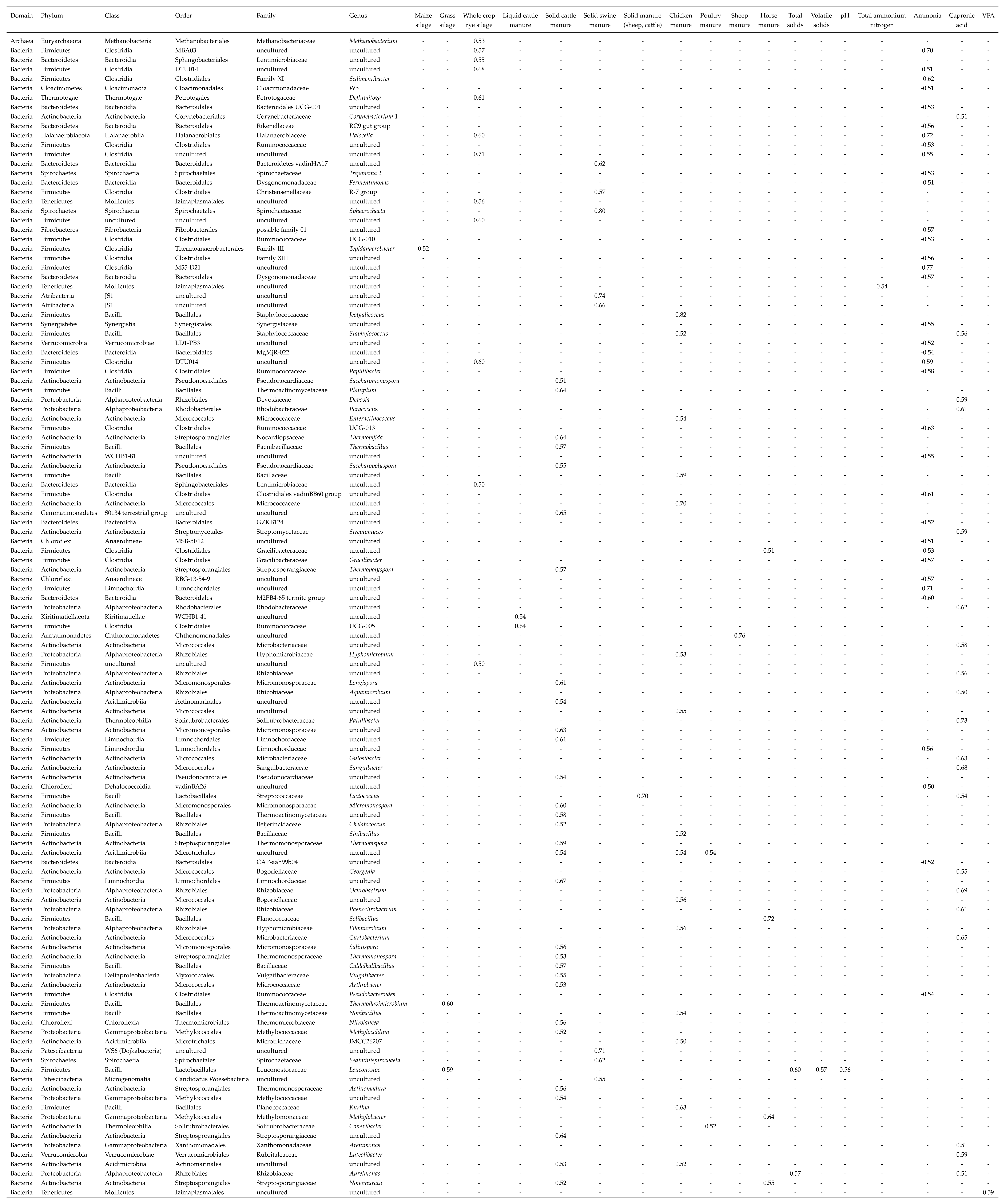

Supplement: Supplementary file 1 [file microorganisms-09-01457-s001.zip › Supplementary_Materials_Proof/Table_S6.png]

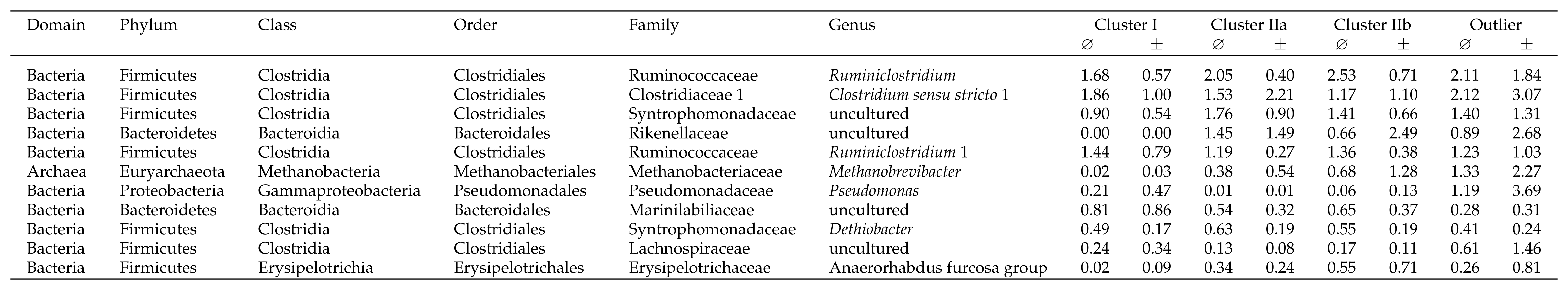

Supplement: Supplementary file 1 [file microorganisms-09-01457-s001.zip › Supplementary_Materials_Proof/Table_S5.png]

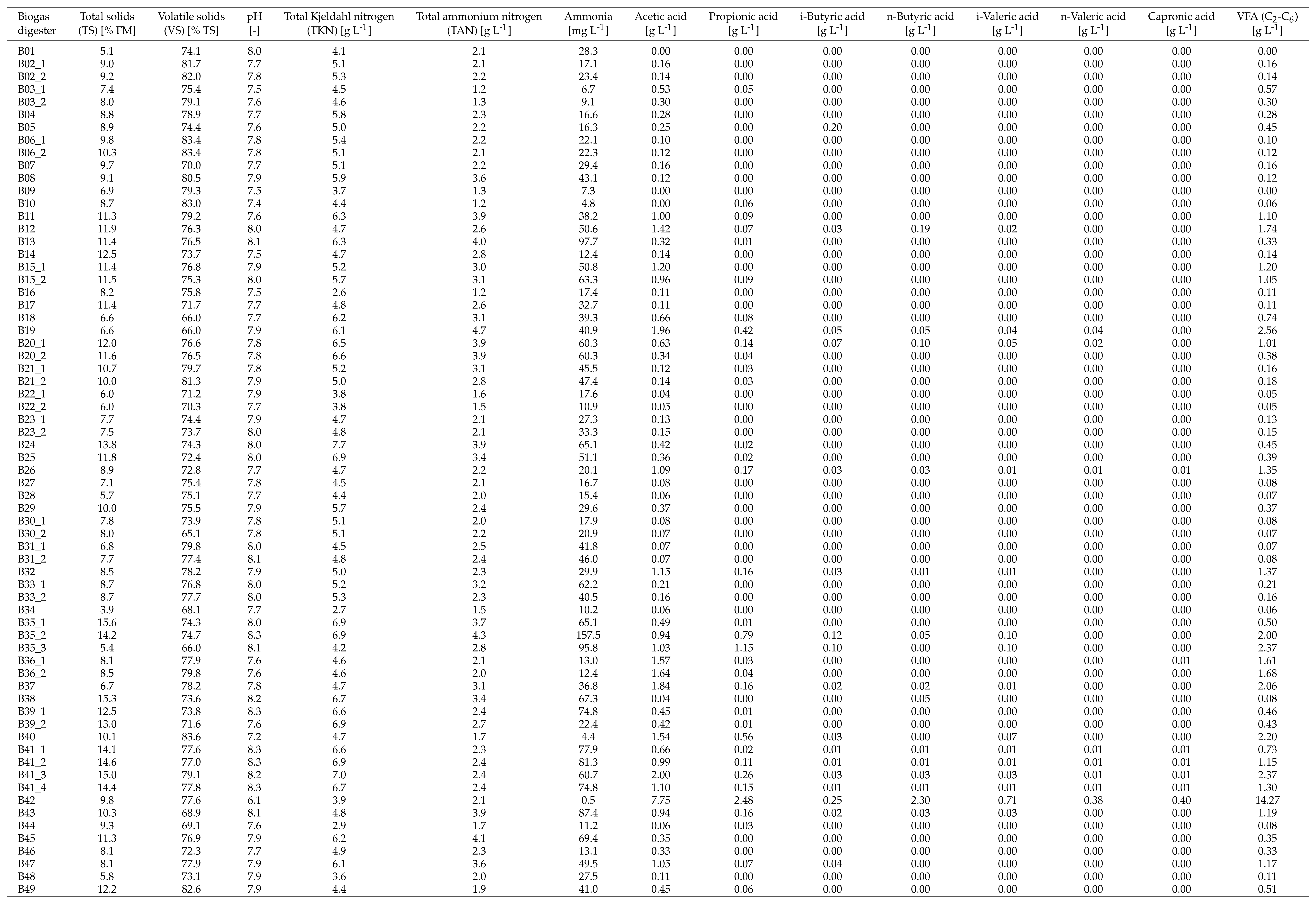

Supplement: Supplementary file 1 [file microorganisms-09-01457-s001.zip › Supplementary_Materials_Proof/Table_S2.png]

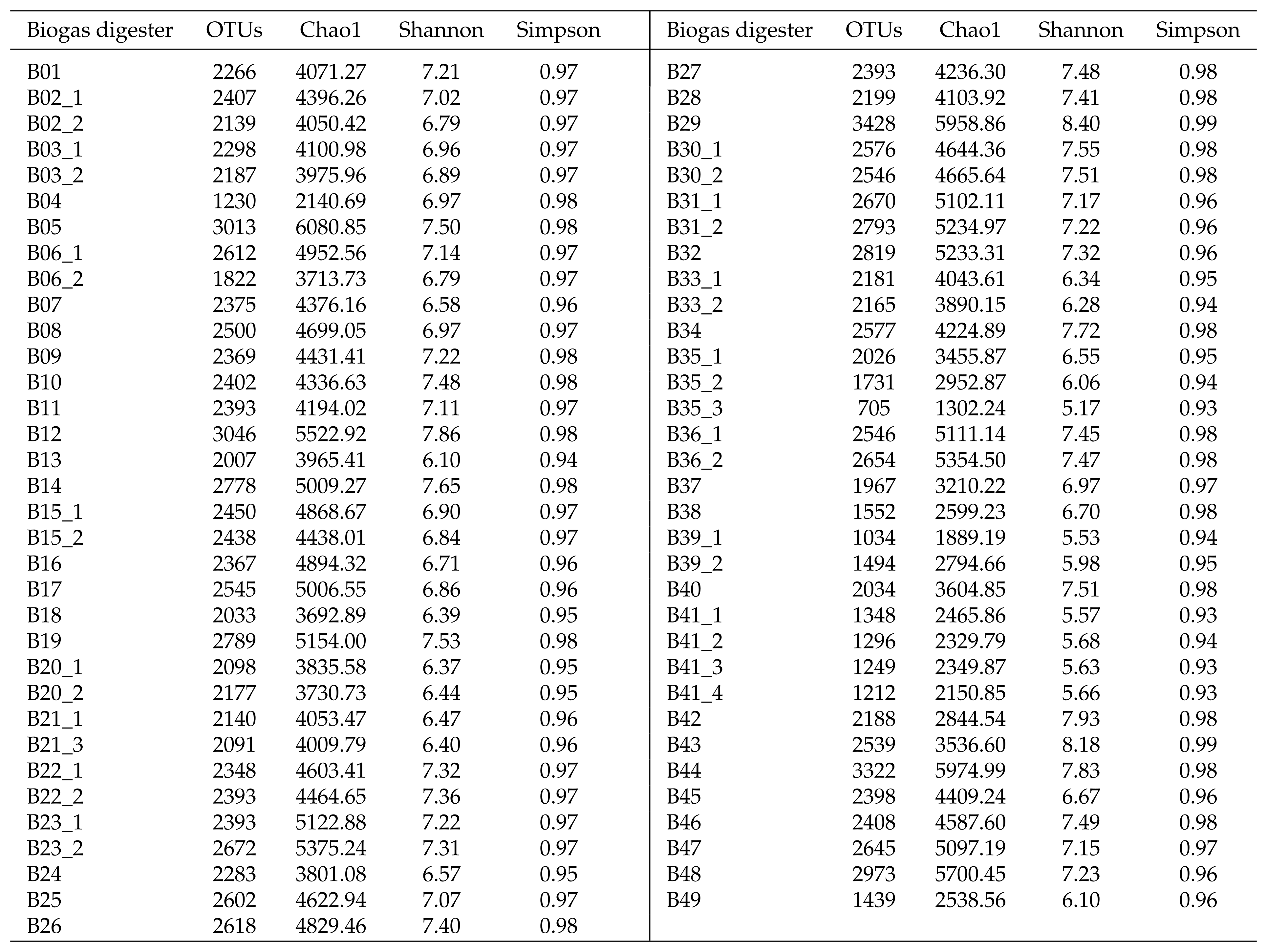

Supplement: Supplementary file 1 [file microorganisms-09-01457-s001.zip › Supplementary_Materials_Proof/Table_S3.png]

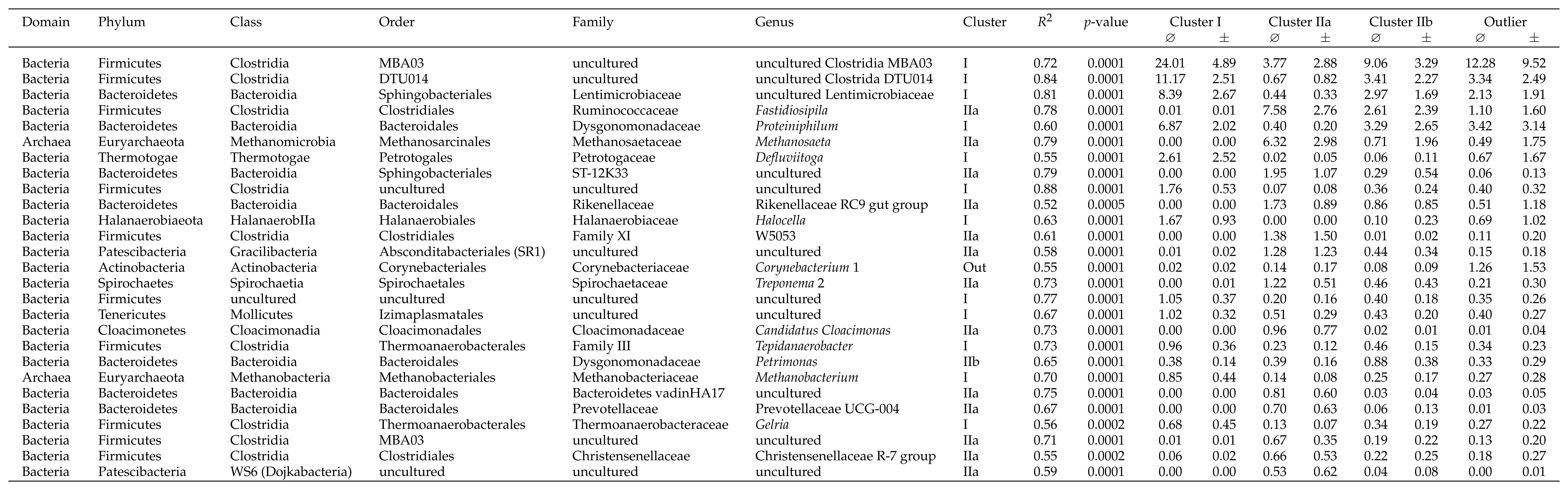

Supplement: Supplementary file 1 [file microorganisms-09-01457-s001.zip › Supplementary_Materials_Proof/Table_S4.png]

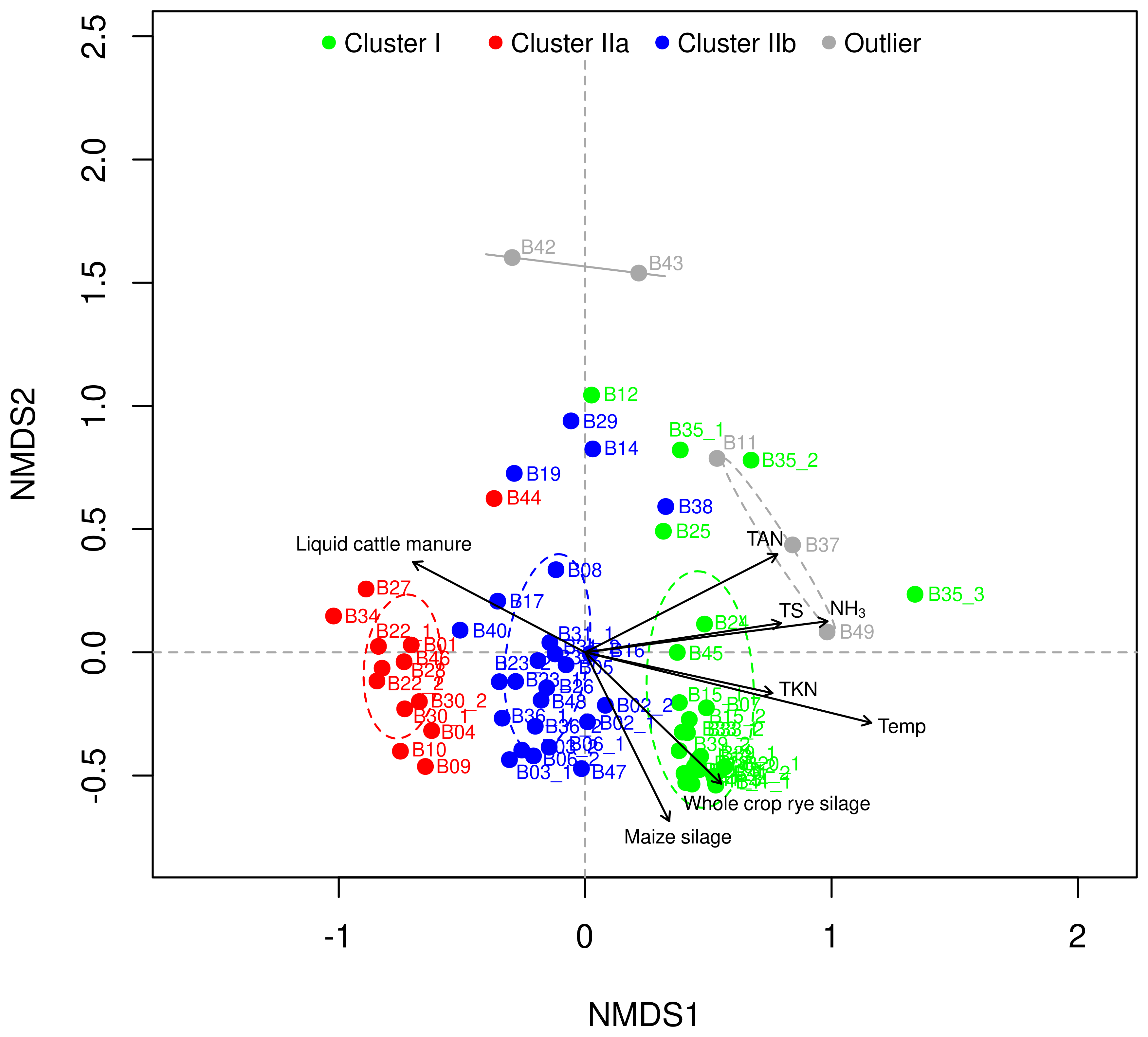

Supplement: Supplementary file 1 [file microorganisms-09-01457-s001.zip › Supplementary_Materials_Proof/Figure_S2.png]
